# Intracranial Aneurysm Rupture Risk Estimation With Multidimensional Feature Fusion

**DOI:** 10.3389/fnins.2022.813056

**Published:** 2022-02-17

**Authors:** Xingwei An, Jiaqian He, Yang Di, Miao Wang, Bin Luo, Ying Huang, Dong Ming

**Affiliations:** ^1^Academy of Medical Engineering and Translational Medicine, Tianjin University, Tianjin, China; ^2^Tianjin Center for Brain Science, Tianjin, China; ^3^Department of Neurosurgery, Huanhu Hospital of Tianjin University, Tianjin, China; ^4^Department of Biomedical Engineering, School of Precision Instruments and Optoelectronics Engineering, Tianjin University, Tianjin, China

**Keywords:** intracranial aneurysm, risk estimation, feature fusion, machine learning, radiomics

## Abstract

The rupture of aneurysms is the main cause of spontaneous subarachnoid hemorrhage (SAH), which is a serious life-threatening disease with high mortality and permanent disability rates. Therefore, it is highly desirable to evaluate the rupture risk of aneurysms. In this study, we proposed a novel semiautomatic prediction model for the rupture risk estimation of aneurysms based on the CADA dataset, including 108 datasets with 125 annotated aneurysms. The model consisted of multidimensional feature fusion, feature selection, and the construction of classification methods. For the multidimensional feature fusion, we extracted four kinds of features and combined them into the feature set, including morphological features, radiomics features, clinical features, and deep learning features. Specifically, we applied the feature extractor 3D EfficientNet-B0 to extract and analyze the classification capabilities of three different deep learning features, namely, no-sigmoid features, sigmoid features, and binarization features. In the experiment, we constructed five distinct classification models, among which the *k*-nearest neighbor classifier showed the best performance for aneurysm rupture risk estimation, reaching an F2-score of 0.789. Our results suggest that the full use of multidimensional feature fusion can improve the performance of aneurysm rupture risk assessment. Compared with other methods, our method achieves the state-of-the-art performance for aneurysm rupture risk assessment methods based on CADA 2020.

## Introduction

An intracranial aneurysm is an abnormal local dilatation of the cerebral artery due to the weakness of the vessel wall. It occurs in approximately 2–5% of the population and is the main cause of non-traumatic subarachnoid hemorrhage (SAH) (Xu et al., 2019). SAH caused by aneurysm rupture is a serious neurological disease with high mortality and morbidity. Despite treatment technology advances and imaging technology improvements currently, the death rate of SAH is approximately 40–50% and leaves approximately half of survivors with permanent neurological deficits (Boulouis et al., 2017; Roked and Reddy, 2020). Therefore, early detection of aneurysms and rupture risk assessment of unruptured aneurysms are clinically significant for the treatment and prognosis of patients.

Aneurysmal morphology such as shape and size, patient-specific clinical factors such as hypertension, smoking, a history of SAH, sex, and population, as well as hemodynamics of aneurysms are known to be risk factors associated with intracranial aneurysm rupture (Abboud et al., 2017; Boulouis et al., 2017). At present, digital subtraction angiography (DSA), computed tomography angiography (CTA), and magnetic resonance angiography (MRA) are primary imaging techniques clinically for rupture risk assessment of aneurysm. Doctors comprehensively assess the rupture risk of aneurysm mainly based on the high-resolution angiographic images and patient-specific clinical factors. However, due to the variations in the level of experience and proficiency among physicians, the evaluation is highly subjective and lacks consistency among experts. Therefore, it is necessary to develop a computer-aided diagnosis system for assessing the rupture risk of the aneurysm to assist doctors in diagnosis and decision-making to avoid overtreatment and risks associated with surgery.

As an important branch of artificial intelligence, machine learning (ML) enables to identify and process complex relationships between features in big data sets and can be rapidly applied to unknown data for prediction (Senders et al., 2018). Some recent studies have shown that ML plays an important role in the rupture risk assessment of aneurysm. Silva et al. (2019) demonstrated the ability of ML to distinguish ruptured and unruptured aneurysms based on conventional radiographic characteristics of aneurysms and patient-specific clinical features. Tanioka et al. (2020) constructed ML classification models for the identification of ruptured aneurysms by applying manually measured morphological variables and hemodynamic parameters. However, for the assessment of the rupture state of aneurysms, incorporating abundant variables into the classification model is the key to affecting the assessment performance.

Radiomics refers to the technology of analyzing and mining high volumes of quantitative features extracted from medical images and then developing a robust model based on the key information that works to support the clinical decision ultimately (Limkin et al., 2017). It has shown considerable potential in many medical challenges, such as auxiliary diagnosis, classification, and grading of diseases (Yun et al., 2019; Peeken et al., 2021). Recently, the application of radiomics combined with ML in the rupture assessment of intracranial aneurysms has shown initial results. A preliminary study (Ou et al., 2021) employed conventional morphological features and radiomics features to construct an ML classification model, which proves the potential use of radiomics signatures in predicting aneurysm rupture. Alwalid et al. (2021) developed a radiomics classification model on CTA images to identify patients with ruptured aneurysms. However, the ability of radiomics features characterizing regions of interest is subject to low-level properties to some extent (Hua et al., 2020). In recent years, deep learning methods, especially convolutional neural networks (CNNs), have achieved excellent results in dealing with the tasks of classification, segmentation, and detection in medical imaging (Zeng et al., 2020; Yang et al., 2021). The convolution and pooling operations in the network automatically learn and capture the local details as well as more complex information and structure features of images, so as to obtain the abstract representation of the image at various scales.

Thus, we deemed that the complementary advantages of deep learning and radiomics technologies could enrich feature representations of medical images and further improve the prediction performance for the rupture risk of the aneurysm. In this study, we explored multidimensional features derived from both high-resolution angiographic images and high-quality three-dimensional aneurysm modeling data to build a semiautomatic prediction model for rupture risk estimation of the aneurysm.

## Materials and Methods

### Dataset

**FIGURE 1:**
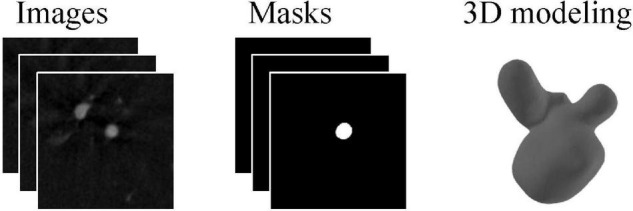
An example case for angiographic image of the aneurysm and corresponding two types of segmentation masks.

The challenge for aneurysm rupture risk estimation is task 3 in cerebral aneurysm detection and analysis (CADA) challenge. The challenge organizers provided 110 datasets with 128 annotated aneurysms. The image data of patients were acquired utilizing the digital subtraction AXIOM Artis C-arm system by a rotational acquisition time of 5 s with 126 frames. Postprocessing was performed using LEONARDO InSpace 3D (Siemens, Forchheim, Germany). All segmentation masks provided by a skilled annotator were checked by an experienced neurosurgeon later. Figure 1 shows the example of the two types of segmentation masks (stereolithography files and image files) for the same aneurysm. In addition, the rupture state and rupture information of each aneurysm are provided. After removing 3 cases with missing information, the remaining 125 cases are included for model training and validation.

### Feature Extraction

In this study, we extracted multidimensional features derived from both angiographic images and three-dimensional aneurysm modeling data, consisting of radiomics features, morphological features, deep learning features, and clinical information. Details are described as follows.

#### Radiomics Features

Before radiomics feature extraction, image preprocessing with intensity normalization to the grayscale range of [0, 100] and isotropic resampling to a uniform pixel dimension of 0.5 × 0.5 × 0.5 mm^3^ was performed. We extracted radiomics features including aneurysm intensity, shape-based, and texture features from regions of interest defined by the angiography images and segmentation masks (image files) using the open-source PyRadiomics package (version 3.0.1) (van Griethuysen et al., 2017). Texture features are visual features that reflect the uniformity of the image and the slow or periodic changes on the surface of the object. Specifically, these extracted characteristics were divided into the following seven groups, including first-order statistics (18 features), shape 3D-based (14 features), gray-level co-occurrence matrix (24 features), gray-level run-length matrix (16 features), gray-level size zone matrix (16 features), neighboring gray-tone difference matrix (5 features), and gray-level dependence matrix (14 features). Most features listed above were in accordance with the recommendations of the Imaging Biomarker Standardization Initiative (IBSI) (Zwanenburg et al., 2020).

#### Morphological Features Based on Stereolithography Files

Currently, shape-based features have shown to be beneficial in assessing the rupture risk of the aneurysm (Abboud et al., 2017; Silva et al., 2019; Tanioka et al., 2020). Therefore, we extracted morphological features of the aneurysm based on the three-dimensional modeling data for a more reliable estimation, including the length, width, height, surface area, and volume. In addition, we considered that curvature features provided additional representations for describing the morphology of aneurysms. The extracted curvature features of aneurysms included the principal curvature, Gaussian curvature, and mean curvature. The maximum, minimum, mean and standard deviation of curvature were calculated, respectively. In this study, 25 morphological features were extracted for each case.

#### Deep Learning Features

**FIGURE 2:**
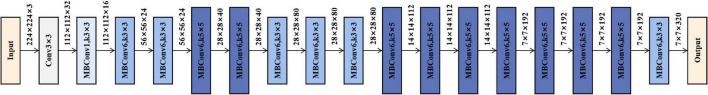
EfficientNet-B0 structure.

**FIGURE 3:**
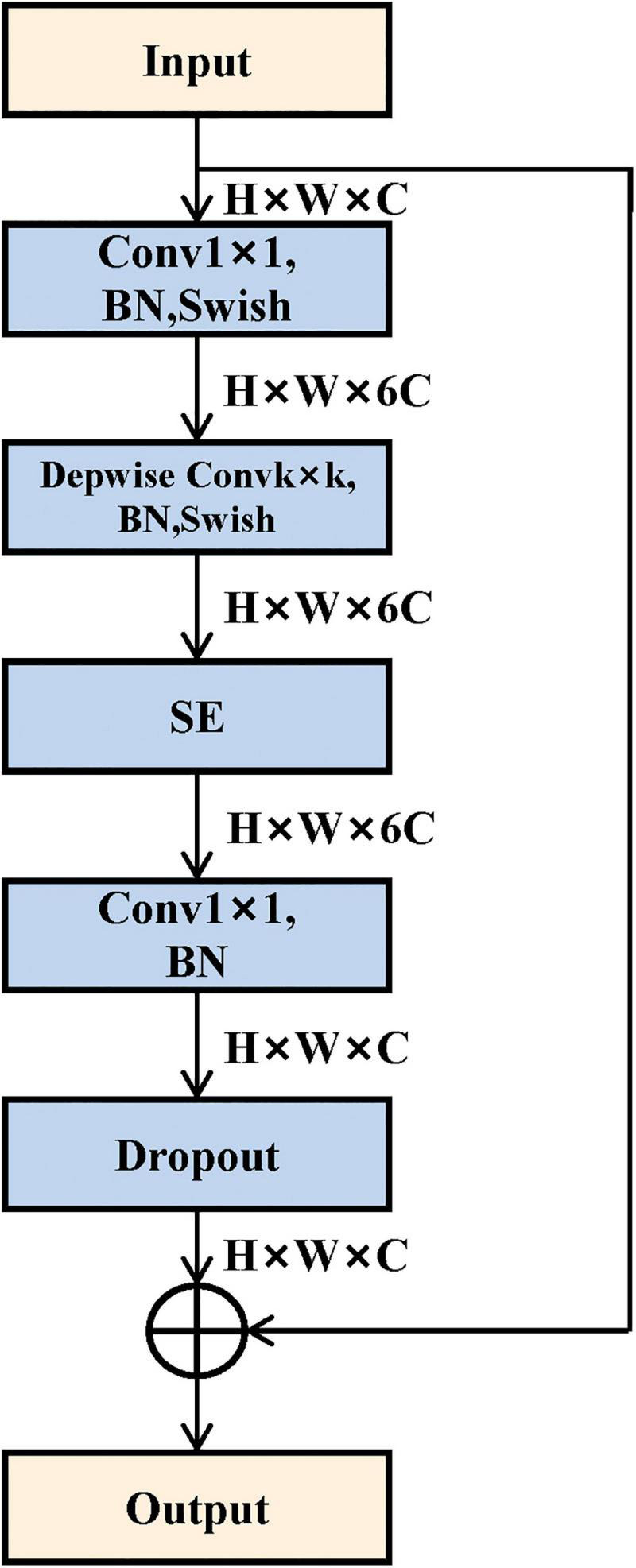
Mobile inverted bottleneck convolution (MBConv) module structure. It mainly consists of depthwise convolution and squeeze-and-excitation block.

To acquire high-level image features, we employed the convolutional neural network method to mine the abstract features. In this study, we selected and trained a 3D EfficientNet-B0 as the feature extractor (Tan and Le, 2019), which balanced the depth, width, and resolution of the model with a highly effective compound coefficient, thereby achieving satisfactory accuracy. Figure 2 shows the network architecture, and its main building block is MBConv (Sandler et al., 2018) with squeeze-and-excitation optimization (Hu et al., 2020), as shown in Figure 3. We, respectively, took the image and mask as the input of the convolutional neural network and explored three various deep learning features from convolutional neural network outputs.

1) No-sigmoid features: outputs of the feature maps only through the final fully connected layer.
2) Sigmoid features: outputs of the feature maps through the final fully connected layer and sigmoid function successively.
3) Binarization features: outputs of the feature maps through the final fully connected layer, sigmoid function, and binarization operation successively. Binarization operation can be calculated as follows:

(1) $$f\,\left(x\right)=\{\begin{array}{c} 1,x\geq 0.5\\ 0,x<0.5 \end{array}$$

**FIGURE 4:**
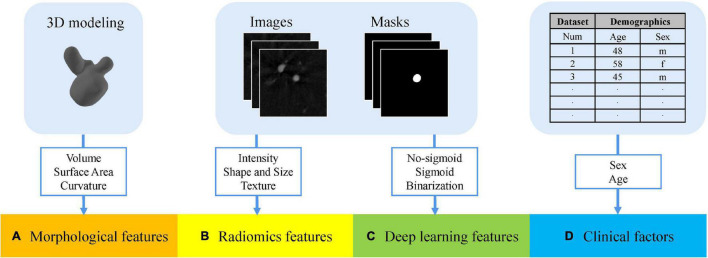
Multidimensional feature set consists of four groups. **(A)** Morphological features extracted from stereolithography files. **(B)** Radiomics features extracted from the angiography images and segmentation masks. **(C)** Deep learning features extracted from the angiography images and segmentation masks. **(D)** Patient-specific clinical factors.

In summary, we combined the multidimensional features above to enrich the feature representation of rupture risk. All the feature sets are shown in Figure 4.

We obtained three types of feature sets as original multidimensional feature sets, namely, no-sigmoid type, sigmoid type, and binarization type. Each original feature set contained four parts of features, which were morphological features (25 features), radiomics features (107 features), the corresponding type of deep learning features (2 features, different feature sets have different deep learning features), and patient-specific clinical factors (2 features, sex, and age). That is, each of the three types of original multidimensional feature sets contained 136 features.

### Nested Cross-Validation

**FIGURE 5:**
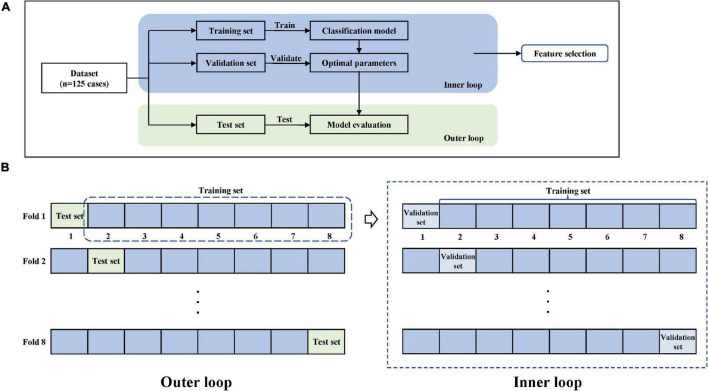
Model training and nested cross-validation. **(A)** General overview. **(B)** 8-fold cross-validation.

Cross-validation can evaluate the generalization ability of ML algorithms to data sets independent of training data and prevent over-fitting effectively (Arlot and Celisse, 2010). Stratified sampling was used in this study to perform cross-validation to ensure that the proportion of samples in each target class in the training set and validation set is the same as the full set. Considering that this traditional cross-validation method cannot solve the problem of optimal model selection and model parameter tuning well, we used nested cross-validation (Varoquaux et al., 2017) in order to search for hyperparameters by estimating the generalization error of the basic model to obtain the best parameters of the model. The process of 8-fold cross-validation is shown in Figure 5, which contains a two-loop nested cross-validation scheme. Hyperparameters were optimized using grid search as part of the inner loop. The optimal hyperparameters were then used for testing on the outer loop.

### Feature Selection

We uniformly standardized all the features by removing the mean and scaling to unit variance. To improve the stability and generalization performance of the model, it is critical to select discriminative features. We chose the random forest (RF) (Genuer et al., 2010) and XGboost (Chen and Guestrin, 2016) methods for feature selection. Both methods could generate a ranking of the feature importance after training and further select some profitable features by setting the threshold. In this study, the following two feature selection steps were applied.

Step 1: We took the features selected simultaneously by RF and XGboost based on the training set of the original multidimensional feature set of each cross-validation fold and merged the features retained by all 8 folds to get the feature set M.

Step 2: We accumulated the important scores of the features in each fold and counted the top 1/2 features of the RF and Xgboost methods in the M set to obtain feature set *M*_r_ and *M*_x_, respectively. *M*_r_ represented feature selection by RF. *M*_x_ represented feature selection by Xgboost. The feature subset *N* was obtained by *N* = *M*_r_∩*M*_x_.

Therefore, the corresponding feature subsets were obtained from the three original feature sets, among which 22 features were retained for the no-sigmoid type feature subset, 24 features for the sigmoid type feature subset, and 24 for the binarization type feature subset. The selected features in the three types of feature subsets are shown in Supplementary Table 1.

### Classification Model

To find an optimal classifier for the classification task of ruptured and unruptured aneurysms, five distinct ML models were used to build the classification model using the selected features, respectively, including support vector machine (SVM) (Cortes and Vapnik, 1995), RF (Breiman, 2001), *k*-nearest neighbor (KNN) (Fix and Hodges, 1989), logistic regression (LR) (Berkson, 1946), and XGBoost (Chen and Guestrin, 2016) classifiers. To ensure the robustness of the experimental results, we adopted 8-fold cross-validation and then took the average of classification metrics as the final result.

### Model Evaluation

Considering that the identification of aneurysms at risk is more important than the avoidance of false-positive risk classification, F2-score was selected as the final score metric in the rupture risk estimation challenge. The F2-score integrates two indicators of recall and precision, and it is considered that recall is twice as important as precision, as shown in Eq. 2. In addition, we also calculated other metrics including accuracy (ACC), the area under the curve (AUC), recall, and precision.


(2) $$F\,2=\frac{5*\mathrm{Precision}*\mathrm{Recall}}{4*\mathrm{Precision}+\mathrm{Recall}}$$

## Results

### Implementation Details

For the EfficientNet-B0 feature extractor, we trained this model on an NVIDIA GeForce RTX 3090 GPU with 24 GB memory. All the images were employed spacing normalization to a common spacing of 0.5 × 0.5 × 0.5 mm^3^ and intensity normalization to the grayscale range of [0, 1]. We resized all the images to 128 × 128 × 128 and set total epochs to 100 for each fold of cross-validation, with the learning rate 3e-4 and batch size 4. The AdamW algorithm was adopted to optimize the feature extraction network. We also used weight decay with 1e-8. Our other experiments were implemented on an AMD Ryzen 5 5600H CPU @3.30 GHz with 16 GB RAM.

### Rupture Risk Estimation Results

After feature selection, we constructed five different classification models with the three feature subsets, and the final results are shown in Table 1. It can be seen that the KNN model based on the sigmoid type feature set achieved the best mean performance on the F2-score. Thus, we chose the sigmoid feature subset as the final feature subset. A heat map was constructed to show the association between selected features and aneurysm rupture status based on the feature subset, as shown in Supplementary Figure 1.

**TABLE 1:** The mean F2-score for different feature sets and classification methods on the test set.

Classifier	Binarization	Sigmoid	No-sigmoid
SVM	**0.730**	0.724	0.609
LR	**0.747**	0.731	0.644
RF	0.695	0.675	**0.707**
XGBoost	0.708	**0.715**	0.698
KNN	0.752	**0.789**	0.580

*The best results for each specified classifier are highlighted in bold red.*

Table 2 shows the comparison of results among different classifiers constructed with this feature set. Based on the F2-score, the KNN model shows the best result with a mean F2-score of 0.789 on the test set. Thus, the KNN algorithm was chosen as the final model. For the presented five classification models, the KNN classifier shows the best performance on most metrics for aneurysm rupture risk estimation. Evaluating the performance of accuracy, the KNN model shows the best result with a mean of 0.791.

**TABLE 2:** Comparison of the results of different classifiers based on the sigmoid feature set.

Classifier	F2-score	ACC	AUC	Precision	Recall
SVM	0.724	0.775	0.820	**0.779**	0.732
LR	0.731	0.776	**0.834**	0.761	0.732
RF	0.675	0.751	0.810	0.771	0.660
XGBoost	0.715	0.767	0.803	0.773	0.714
KNN	**0.789**	**0.791**	0.811	0.755	**0.803**

*The best results for each of these metrics are highlighted in bold red.*

**FIGURE 6:**
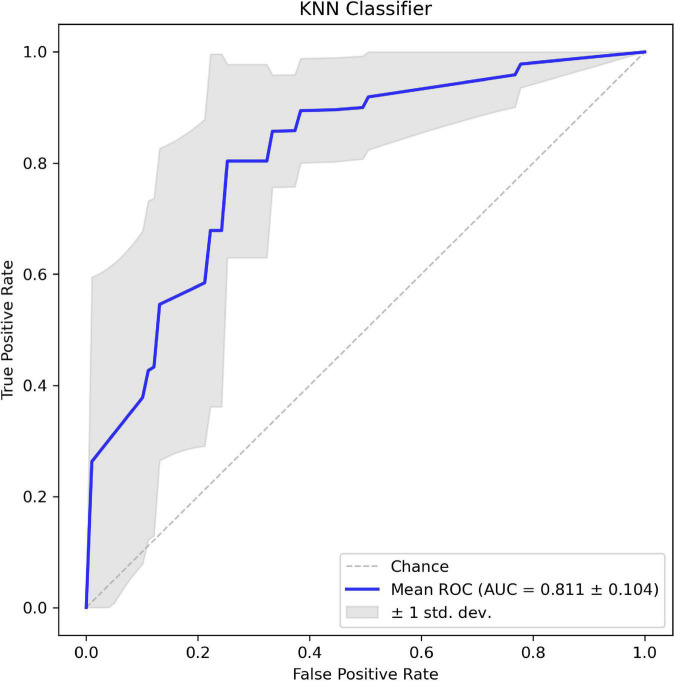
The mean receiver operating characteristic (ROC) curve of the *k*-nearest neighbor (KNN) classifier based on 8-fold cross-validation.

In addition, the corresponding mean receiver operating characteristic (ROC) curve over all outer folds based on the optimal model is shown in Figure 6. The KNN model shows a good performance in the classification of aneurysm rupture with a mean AUC of 0.811 on the test set.

Ivantsits et al. (2021) and Liu et al. (2021), respectively, proposed two excellent semiautomatic aneurysm rupture risk estimation methods on the CADA dataset. Table 3 shows the comparison on the metrics of our approach with two related works. It is observed that our approach achieved better performance than both related works on the CADA dataset. Under the condition of using the same classifier, our methods performed better, which proves that the features we extracted were more suitable and effective for this task.

**TABLE 3:** Aneurysm rupture risk estimation performance of our method and two related works based on CADA dataset.

Classifier	Method	F2-score	ACC	AUC	Precision	Recall
XGBoost	Liu et al., 2021	0.673	0.652	n/a	0.583	0.700
	Ours	**0.715**	**0.767**	**0.803**	**0.773**	**0.714**
KNN	Ivantsits et al., 2021	0.690	0.660	n/a	n/a	n/a
	Ours	**0.789**	**0.791**	**0.811**	**0.755**	**0.803**
RF	Ivantsits et al., 2021	n/a	0.690	n/a	n/a	n/a
	Ours	**0.675**	**0.751**	**0.810**	0.771	**0.660**
SVM	Ours	0.724	0.775	0.820	**0.779**	0.732
LR	Ours	0.731	0.776	**0.834**	0.761	0.732

*Better results for each specified classifier are highlighted in bold black. The best results for each of these metrics are highlighted in bold red.*

## Discussion

Intracranial aneurysm rupture is a catastrophic medical event with high mortality and permanent disability risk. A timely and accurate rupture risk estimation of aneurysms is necessary for clinical treatment. At present, the widespread availability of vascular neuroimaging has allowed more unruptured aneurysms to be discovered incidentally, but the treatment decision-making for aneurysms is still a challenge that the clinic needs to face because doctors are required to weigh the risk of SAH along with the risks of surgical or endovascular treatments and subsequent complications with discretion (Boulouis et al., 2017).

The morphology of the intracranial aneurysm is considered to be associated with the rupture state of the aneurysm (Abboud et al., 2017; Boulouis et al., 2017). Most of the previous reports employed manually measured morphological indicators to identify the rupture risk (Liu et al., 2018; Silva et al., 2019; Tanioka et al., 2020), which did not fully explore the rich information of angiography images.

In this study, we proposed a classification method based on diverse types of risk factors for the assessment of aneurysm rupture state, so as to promote timely management of patients and provide some guidance for follow-up treatment decisions. In the pipeline for assessing the rupture risk of aneurysm, our proposed method consisted of multidimensional feature fusion, feature selection to capture the discriminative variables, and followed by the construction of classification models. We combined multidimensional feature representations related to rupture risk factors of aneurysms including radiomics features, morphological features, deep learning features, and patient-specific clinical factors. Considering the powerful feature extraction capability of deep learning for images, we took the deep learning network as a feature extractor to extract and analyze the classification capability of three different deep learning features. The results indicate a great potential of the sigmoid type feature subset as a risk factor for intracranial aneurysm rupture estimation.

The sigmoid type feature subset included deep learning descriptors, shape descriptors, first-order histogram descriptors, and texture descriptors. As high-level semantic features, the deep learning features proposed in this study could learn complex information patterns and structural features in image data, which are invisible to human eyes. Curvature features represented as additional morphological features may reflect changes related to the aneurysm rupture state. Radiomics features are calculated in a pixel-by-pixel manner, which can quantitatively describe the morphology of the 3D lesion. In this study, nine radiomics features were finally retained. It proves the potential of radiomics features for the classification of aneurysm rupture, which is consistent with previous studies (Alwalid et al., 2021; Ou et al., 2021). Texture patterns within the aneurysm region especially the aneurysmal lumen may be caused by the uneven distribution of contrast agents, which were thought to be related to turbulence flow (Ou et al., 2021). It is generally considered that turbulent flow could activate inflammatory mechanisms and could be associated with higher-risk lesions (George et al., 2016). This further explains why texture features could be used as the risk factor for assessing aneurysm rupture. For clinical variables, both sex and age were not included in the final feature subset. It could be due to its complicated mechanism on aneurysm rupture and the experiment being based on a small data set, and further studies are needed to prove the relationship between clinical variables and rupture outcome.

Our study has some limitations that are worth noting. One is that it takes some computational cost to extract deep learning features due to the large size of angiographic images. The other is that due to the limited amount of data provided, further verification is required on external data. In the future, we plan to collect clinical data to verify the robustness of our approach and take steps to further optimize the performance of our model to achieve a more efficient automatic aneurysm rupture risk assessment. Recent studies have shown that computer-aided diagnosis algorithms for aneurysm detection have the potential to shorten reading times and enhance the performances of radiologists (Shi et al., 2020). A further idea is considered to effectively integrate this work with aneurysm detection to build a complete automatic aneurysm diagnosis system, which may improve efficiency in the radiology department (Alwalid et al., 2021) and promote timely management for patients.

## Conclusion

In this study, we assumed that multidimensional feature fusion and feature selection strategies are necessary to enhance the level of aneurysm rupture risk assessment. Based on the inspiration, we combined morphological features, radiomics features, clinical features, and deep learning features with the feature extractor 3D EfficientNet-B0 to propose a novel semiautomatic ML algorithm for aneurysm rupture risk assessment. Our results demonstrate that the multidimensional risk factors we proposed could improve the ability to identify the ruptured state of the aneurysm. Compared with other methods, our method outperforms the state-of-the-art aneurysm rupture risk assessment method based on CADA 2020, which shows the good prospect of application in decision support systems for patients with aneurysms.

## Data Availability Statement

Publicly available datasets were analyzed in this study. It can be found at CADA Rupture Risk Estimation Challenge: https://cada-rre.grand-challenge.org.

## Author Contributions

DM and YH contributed to the conception and design of the study. JH, YD, and MW performed data analysis. JH and XA drafted the manuscript. XA edited the manuscript and supervised the entire study. BL interpreted the annotated data. All authors contributed to the study and approved the submitted version.

## Conflict of Interest

The authors declare that the research was conducted in the absence of any commercial or financial relationships that could be construed as a potential conflict of interest.

## Publisher’s Note

All claims expressed in this article are solely those of the authors and do not necessarily represent those of their affiliated organizations, or those of the publisher, the editors and the reviewers. Any product that may be evaluated in this article, or claim that may be made by its manufacturer, is not guaranteed or endorsed by the publisher.
