# Supplementary material for: Intracranial Aneurysm Rupture Risk Estimation With Multidimensional Feature Fusion
Source: Front Neurosci. 2022 Feb 17;16:813056. doi: 10.3389/fnins.2022.813056 (PMC8893318; doi:10.3389/fnins.2022.813056)

Supplementary Material

Supplementary Figure 1. The heatmap of final features selected (sigmoid type feature subset) for aneurysm rupture risk estimation. Each row and column correspond to one aneurysm and one standardized feature, respectively. The dendrogram shows the hierarchical clustering of features.

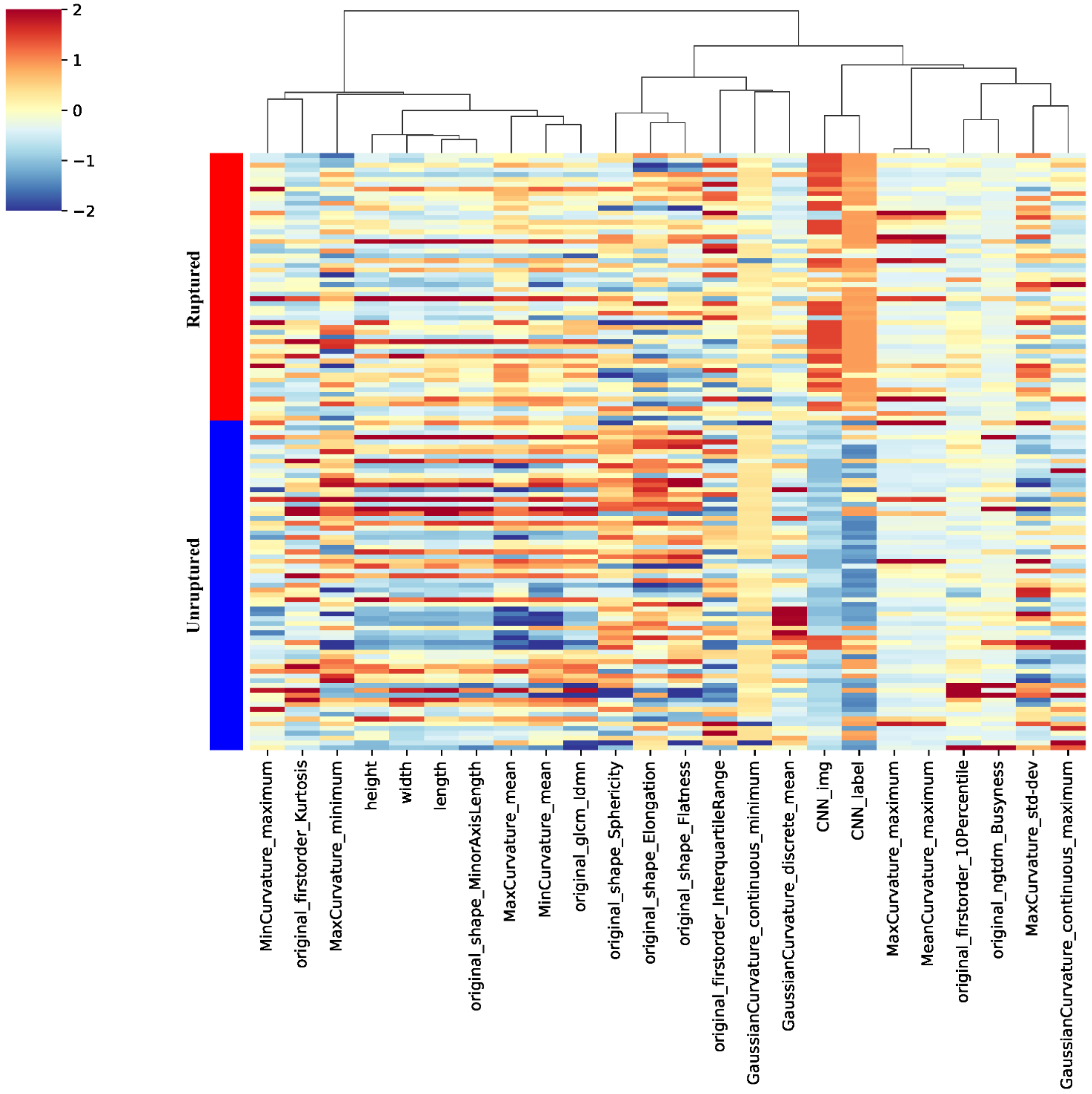

Supplement: Supplementary file 1 [file Image_1.pdf]
