# Supplementary material for: Intracranial Aneurysm Rupture Risk Estimation With Multidimensional Feature Fusion
Source: Front Neurosci. 2022 Feb 17;16:813056. doi: 10.3389/fnins.2022.813056 (PMC8893318; doi:10.3389/fnins.2022.813056)
Supplement: Supplementary file 2 [file Table_1.pdf]

Supplementary Material

Supplementary Table 1. The selected features in three types of feature subsets. Dlf, deep learning features; Cf, clinical factors; Mpf, morphological features; Raf, radiomics features.

Feature subset	Feature	Feature class
No-sigmoid type feature subset (22 features)	CNN_img	Dlf
	CNN_label	Dlf
	width	Mpf
	height	Mpf
	length	Mpf
	MinCurvature_mean	Mpf
	MinCurvature_maximum	Mpf
	MaxCurvature_mean	Mpf
	MaxCurvature_std-dev	Mpf
	MaxCurvature_maximum	Mpf
	MaxCurvature_minimum	Mpf
	MeanCurvature_maximum	Mpf
	GaussianCurvature_continuous_maximum	Mpf
	GaussianCurvature_continuous_minimum	Mpf
	GaussianCurvature_discrete_mean	Mpf
	original_firstorder_10Percentile	Raf
	original_firstorder_InterquartileRange	Raf
	original_glcm_Idmn	Raf
	original_shape_Elongation	Raf
	original_shape_Flatness	Raf
	original_shape_Maximum2DDiameterRow	Raf
	original_shape_Sphericity	Raf
Sigmoid type feature subset (24 features)	CNN_img	Dlf
	CNN_label	Dlf
	width	Mpf
	height	Mpf
	length	Mpf
	MinCurvature_mean	Mpf
	MinCurvature_maximum	Mpf
	MaxCurvature_mean	Mpf
	MaxCurvature_std-dev	Mpf
	MaxCurvature_maximum	Mpf
	MaxCurvature_minimum	Mpf
	MeanCurvature_maximum	Mpf

	GaussianCurvature_continuous_maximum	Mpf
	GaussianCurvature_continuous_minimum	Mpf
	GaussianCurvature_discrete_mean	Mpf
	original_firstorder_10Percentile	Raf
	original_firstorder_InterquartileRange	Raf
	original_firstorder_Kurtosis	Raf
	original_glcm_Idmn	Raf
	original_ngtdm_Busyness	Raf
	original_shape_Elongation	Raf
	original_shape_Flatness	Raf
	original_shape_MinorAxisLength	Raf
	original_shape_Sphericity	Raf
Binarization type feature subset (24 features)	CNN_img	Dlf
	CNN_label	Dlf
	age	Cf
	width	Mpf
	height	Mpf
	length	Mpf
	MinCurvature_maximum	Mpf
	MaxCurvature_mean	Mpf
	MaxCurvature_maximum	Mpf
	MaxCurvature_minimum	Mpf
	MeanCurvature_mean	Mpf
	MeanCurvature_maximum	Mpf
	GaussianCurvature_continuous_mean	Mpf
	GaussianCurvature_continuous_maximum	Mpf
	GaussianCurvature_continuous_minimum	Mpf
	GaussianCurvature_discrete_maximum	Mpf
	original_firstorder_10Percentile	Raf
	original_firstorder_InterquartileRange	Raf
	original_ngtdm_Busyness	Raf
	original_ngtdm_Coarseness	Raf
	original_shape_Elongation	Raf
	original_shape_Flatness	Raf
	original_shape_MinorAxisLength	Raf
	original_shape_Sphericity	Raf
